# Graphene Encapsulated Al Particles for Improvement of Thermal Conductivity in Composites

**DOI:** 10.3390/ma13163602

**Published:** 2020-08-14

**Authors:** Jinuk Hwang, Woo-Seong Tak, So Youn Mun, Sangyong Nam, Sook Young Moon, Woo Sik Kim

**Affiliations:** 1 Fibrous Ceramics and Aerospace Materials Center, Korea Institute of Ceramic Science and Technology, Jinju-si 52851, Korea; ghkdwlsdnr11@gmail.com (J.H.); tws1206@kicet.re.kr (W.-S.T.); msy@kicet.re.kr (S.Y.M.); 2 Department of Materials Engineering and Convergence Technology, Engineering Research Institute, Gyeongsang National University, Jinju-si 52851, Korea; walden@gnu.ac.kr; 3 Institute of Advanced Composite Materials, Korea Institute of Science and Technology (KIST), 55324 Wanju-gun, Korea; moon.sookyoung@kist.re.kr

**Keywords:** graphene reinforced aluminum matrix composites, self-assembly coating, thermal conductivity, uniform dispersion

## Abstract

Graphene reinforced aluminum matrix composites (GRAMCs) with improved thermal conductivity were prepared via a pH-controlled self-assembly process that involved adjusting the concentration of dispersed graphene oxide (GO) solutions. Uniform dispersion was achieved using GO coating on the aluminum (Al) surfaces. Graphene encapsulated Al powders (Al/GO) were sintered through spark plasma sintering (SPS) to prepare bulk composites, these were then analyzed to determine the thermal and mechanical properties. The density of the Al/GO composites was determined to be 99% or more compared to the theoretical density of pure Al. The Vicker’s hardness and thermal conductivity increased by about 47% and 15% more than the pristine Al bulks. These processes can improve properties of the thermal interface between GO and Al, enabling uniform coating without a crosslinking agent. An Al/GO composite, fabricated through the pH-controlled self-assembly process, should be useful for various applications requiring to high thermal conductivity.

## 1. Introduction

Portable electronic devices exhibit continuous demand for light weight, miniaturization and high performance. As the performance of electronic devices improves, more thermal energy is generated in the devices. Failure to efficiently remove the generated heat can cause problems such as reduced performance and lifetime, and increased failure rate of electronic devices [1,2].

The theoretical thermal conductivity of aluminum (Al) is about 230 W/mK, which is 60% of the theoretical thermal conductivity of copper (Cu, 400 W/mK). However, Al has a density (2.70 g/cm^3^) lower than that of copper, so it is more suitable than other metal materials as a heat dissipation material for small and lightweight electronic devices [3,4].

Nano carbon materials, such as graphite, carbon nanotubes (CNTs) and graphene, have a hexagonal conjugated structure with carbon atoms and therefore extremely good mechanical strength and thermal and electrical properties [5,6]. Graphene is a two-dimensional nano carbon material with sp2-bonded carbon atoms it has a theoretical thermal conductivity of 5000 W/mK [7], a very large specific surface area, and it can be combined with various functional materials. Therefore, graphene has various advantages in processes involving dispersing and bonding with matrix materials. It is also used as an additive to improve the mechanical, electrical and thermal properties of composite materials [8,9,10].

Much research on graphene reinforced aluminum matrix composites (GRAMCs) has been conducted to improve the low mechanical strength and the thermal and electrical properties of raw Al. The uniform dispersion of fillers and good interfacial interaction between additives and the matrix materials are important factors influencing the enhancement of characteristics by combining with the Al matrix and carbon fillers [11,12,13].

In order to further improve the properties of the composite material, many studies attempted to achieve homogeneous dispersion of graphene. However, graphene has very large specific surface area, which can be easily agglomerated by van der Waals forces [14]. In addition, due to low interface bonding between graphene and the passivation oxide layer on the aluminum surface, it is difficult to manufacture excellent GRAMCs [15,16].

The traditional ball-milling method has been the most common choice to attempt homogeneous dispersion of graphene. Nevertheless, complete dispersion of nano-sized graphene was not achived. The weak mechanical strength of Al can lead it to form a plate shape during the ball milling process, which may hinder densification. Lin Jiang et al. [17]. achieved a uniform dispersion by coating oxidized CNT, via surface modification of Al, with poly vinyl alcohol (PVA). Because its coating method led to more homogeneous dispersion than that possible with the ball-milling method, Al/PVA/CNT has been found to have a higher density and tensile strength than those with Al/CNT mixed through. To improve dispersibility, A.F. Boostani et al. [18] synthesized various types of hybrid graphene nano sheets (GNSs) additives through graphene encapsulation, after agglomeration of SiC nanoparticles. These were added to the Al matrix to prove the effect by calculating the tensile and elongation rates through a mathematical model.

Chuangan et al. [19]. generated electrons from activated metal surfaces under acidic pH conditions to study the reduction and deposition of graphene oxide (GO). They confirmed that using this mechanism, reduced GO (rGO) was uniformly coated on the surface of individual metal particles. It has been reported that composite particles coated with rGO have high oxidative stability due to excellent bonding between rGO and the metal surfaces. Additionally, acidic solutions allow for the removal and suppression of oxide films present on the metal surface. This can be used eliminate the Al_2_O_3_ layer that causes de-bonding, which can be expected to improve the thermal conductivity of Al-nano carbon composites [20,21,22].

In this study, GRAMCs with improved dispersibility and thermal conductivity were fabricated by GO coating on Al surfaces. The oxide layer on the surface of the Al particles was removed using the acidity of the GO solution, dispersed in water. Then, GO was coated on Al through a pH-controlled self-assembly reaction between activated Al powders and dispersed GO solution. Graphene encapsulated Al powder (Al/GO) composites were prepared without Al_4_C_3_ production by using the SPS to perform sintering for a short time at 600 °C. Measurement of the self-assembled GRAMCs was carried out to determine the thermal and mechanical properties according to the variation of GO contents in the Al matrix.

## 2. Materials and Methods

### 2.1. Synthesis of GRAMCs by GO Coated on Al Surfaces

**Figure 1:**
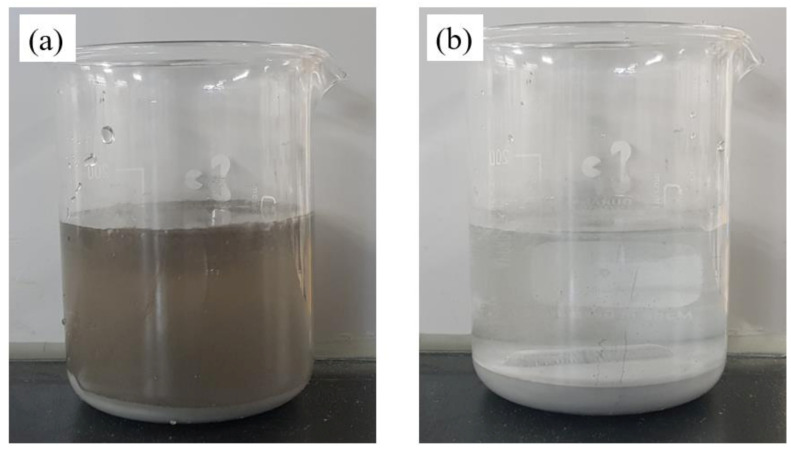
Difference in colors of solution (**a**) before coating of graphene oxide (GO) on aluminum (Al) particles (brown solution) and (**b**) after deposition of Graphene encapsulated Al powder (Al/GO) composites particles (clear solution).

Spherical Al particles (99.5%, Sigma Aldrich, St. Louis, Missouri, USA) with 44 µm diameter, and commercially produced GO (Grapheneall, Siheung, Korea) were used as raw materials for this experiment. The GO dispersed solution was adjusted to 3.0 pH by adjusting the GO distilled water concentration by carboxyl acid groups of GO. The acidic solution was treated with a tip-ultrasonicator at 300 W for 1 h to make a solution with evenly distributed GO. The finished GO solution was transferred to a beaker to be 0.1 wt.%–0.6 wt.% based on the weight of Al, and then stirred continuously to prevent the GO from sinking. Ten g of aluminum was added to each stirred solution and reacted at a temperature of 60 °C for 1 h. After completing the reaction for 1 h, it was confirmed that the color of GO solution became completely transparent, from the previous brown color (Figure 1a). After the coating process, the Al/GO particles were separated from the solution through vacuum filtration to remove the remaining GO in the solution. The powders were then dried over night at 60 °C in a vacuum oven. One g of the fully-dried Al/GO was inserted into a graphite mold with a diameter of 12.5 mm. Then, sintering was performed for 5 min while applying a pressure of 50 MPa at a temperature of 600 °C using SPS (heating rate: 100 °C/min). The sintered samples by SPS were heat treated for 3 h at 550 °C in a pressure-less furnace to remove thermal and residual stresses with nitrogen atmosphere.

### 2.2. Characterizations

The Al/GO particles prepared through the self-assembly reaction between GO solution and Al powder were analyzed by field emission-scanning electron microscopy (FE-SEM, JSM-6700F, JEOL, Tokyo, Japan) to the determine the microstructure. Raman spectroscopy (inVia Ramanmicroscope 514 nm, Renishaw, Wotton-under-Edge, UK) and X-ray photoelectron spectrometer (XPS, Thermoscientific-Nexsa, Waltham, MA, USA) analyses were performed to confirm the mechanism of reduction and deposition of GO on the surface-charged Al particles in acidic solution. The presence or absence of Al_4_C_3_ formation in the sample was confirmed through X-ray diffraction (XRD, D-max 2500, Rigaku, Tokyo, Japan) analysis. Mechanical properties, density and Vicker’s hardness (ZHV 30, Zwick Roell, Ulm, Germany) of the samples were measured. The density of bulk composites was measured by using Archimedes principle to calculate the values measured by the hydrometer. Then, thermal properties of Al/GO, thermal diffusivity and specific heat were analyzed by the laser flash method (LFA-427, NETZSCH, Selb, Germany).

## 3. Results

**Figure 2:**
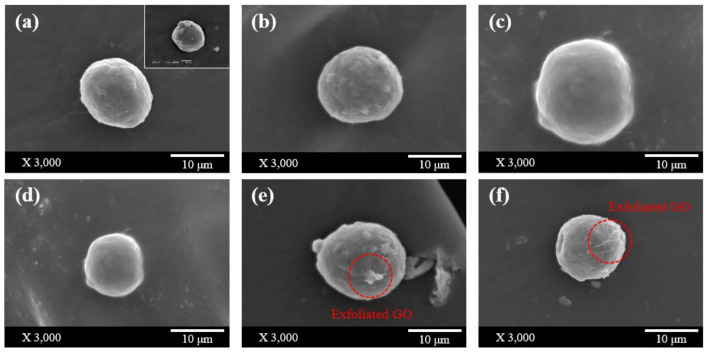
SEM images of (**a**) Al/GO 0.1 wt.% and pristine Al particle (inset image), (**b**–**f**) Al/GO 0.2 wt.%–0.6 wt.%.

Microstructures of Al/GO particles having various GO contents prepared through the self-assembly method were compared through FE-SEM analysis. (Figure 2) It was confirmed that GO was uniformly coated on the surface of the Al particles at levels of GO content up to 0.4 wt% (Figure 2a–d). However, when 0.5 wt.% of GO or more was used, it was confirmed that GO peeled off the surface of the Al particles (Figure 2e,f). The exfoliated GO was able to form aggregations inside the sintered composites, and there is a possibility that this reduced the mechanical and thermal properties of the composite material.

**Figure 3:**
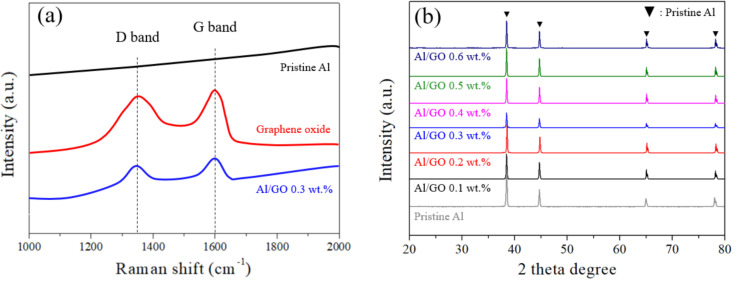
Comparison of (**a**) Raman spectra of bare Al, GO and Al/GO 0.3wt.% powder and (**b**) XRD patterns of sintered Al/GO composites for verity of GO contents.

To confirm the presence of GO coating on the surface of the Al particles, Figure 3a shows a comparison of Raman spectra of the pristine Al, GO and Al/GO 0.3 wt.%. Raman spectra were measured in the range of 1000 to 2000 cm^−1^. No peak of pristine Al powder was observed in the range of measurement, but peaks of the GO and Al/GO 0.3 wt.% samples were identified and had the same values as that of GO in the G band (1580 cm^−1^), showing sp^2^ binding, a D band (1350 cm^−1^) was also generated by sp^3^ bonding of graphene [23].

The formation of Al_4_C_3_ at the interface of Al and graphene is known to decrease the mechanical, thermal and electrical properties of GRAMCs. Al_4_C_3_ is a brittle compound that can be produced at the interface of Al and carbon fillers. Excess Al_4_C_3_ severely weakens the interface bond strength, and the interface is susceptible to corrosion by water. Due to these characteristics, it is possible to decrease the corrosion resistance and properties of the final composites [24]. Densification using SPS was able to prevent synthesis of the Al_4_C_3_ phase by sintering the Al and carbon materials in a short period of time [25]. Through XRD analysis, it can be seen that the Al_4_C_3_ phase did not form in any of the GRAMCs produced using various amounts of GO content in the Al matrix (Figure 3b) [26].

**Figure 4:**
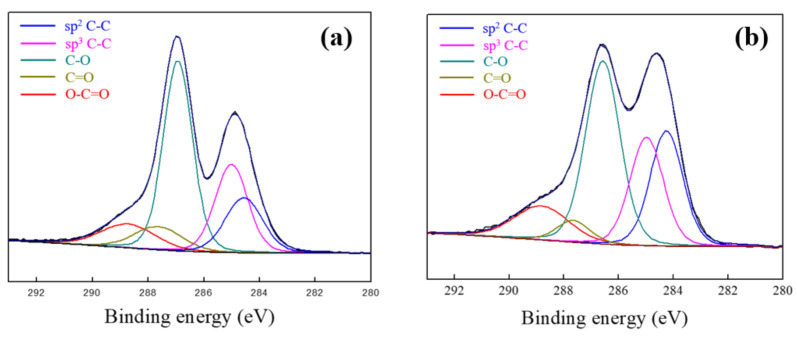
C1s peaks of XPS spectrum: (**a**) graphene oxide and (**b**) Al/GO 0.3wt.%.

The binding energy of sp2 C-C (284 eV) and sp3 C-C (285 eV), due to the structure of graphene, can be clearly seen in the C1s peaks of GO (Figure 4a) and Al/GO 0.3 wt.% (Figure 4b), analyzed by high resolution XPS. In addition, peaks of C-O (286 eV) and O-C-O (288 eV), of the oxygen containing-functional groups of the samples, were confirmed [27,28]. Table 1 shows all of the components of the high-resolution XPS spectrum (Figure 4). The fabrication of Al/GO generates electrons on the Al surface where the oxide layer is removed by the acidity of the GO solution. GO in solution reacts with the generated electrons from the Al surface and is coated on the Al particles through reduction and deposition. The C1s peak of the GO revealed a ratio of carbon–carbon bondings (sp2 C-C and sp3 C-C) to carbon–oxygen bondings (C-O, C=O and O-C=O) at 38.31:61.69. On the other hand, C1s peak of the Al/GO 0.3 wt.% showed a ratio of 45.23:54.77. Through XPS results, it was proven that GO was coated on the Al surface by reduction and deposition between different materials.

**Figure 5:**
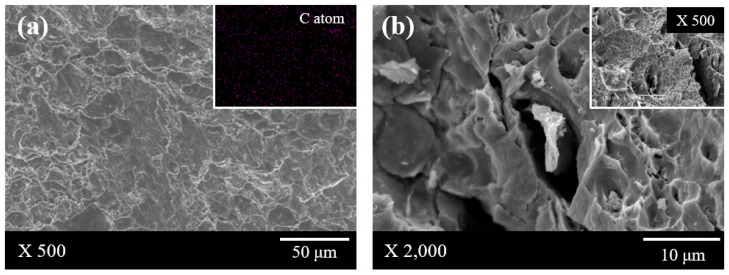
Cross section SEM images of Graphene reinforced aluminum matrix composites (GRAMCs) fabricated by (**a**) self-assembly coating and (**b**) ball- milling mixing method.

Figure 5 shows cross-section SEM images of Al/GO composites prepared by the self-assemble coating and ball-milling method. In Figure 5a, the graphene was well dispersed in the composite material, so aggregated graphene was not observed. In Figure 5b, agglomerated graphene and a number of micro-scale pores were observed (Figure 5b inset). Micro pores of composites degrade the thermal and mechanical properties of GRAMCs. Therefore, graphene must be uniformly dispersed in the Al matrix in order to improve their properties of Al/GO composite. It was confirmed that graphene can be homogeneously distributed in the matrix material through the use of the hybrid materials coated with GO on Al particles.

**Figure 6:**
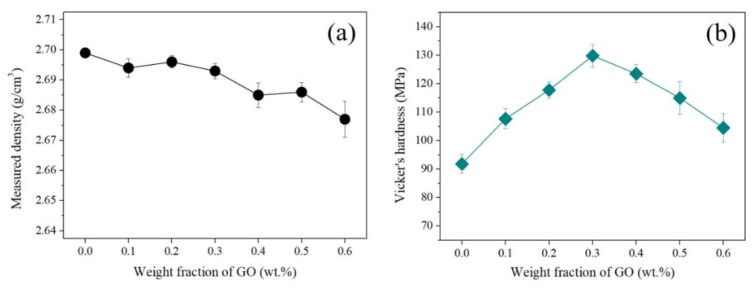
Mechanical properties of composites by weight fraction of GO: (**a**) density measured using Archimedes principles and (**b**) Vicker’s hardness.

Table 2. shows the mechanical and thermal properties of all the Al/GO composites prepared through the SPS process. Density of the sintered samples (Figure 6a) decreased with the increase in GO content. It is known that if graphene is not uniformly dispersed in the matrix, density is lowed. However, the density of GRAMCs in this research is close to the theoretical density of pure Al with a relative density of over 99%. If graphene is not uniformly dispersed, they are agglomerated to clusters in the Al matrix. The aggregated clusters reduce density through closed pore formation in sintered Al, and finally decease the properties of composites [29,30]. The density of our composites can demonstrate the uniform dispersion of GO in the matrix, higher density by homogeneously dispersed nano-carbon additives.

The addition of graphene is known to increase the hardness by providing resistance to deformation when the complex is under pressure. Graphene addition is directly affected by the degree of dispersion of graphene and the interfacial properties of the matrix and additives. In addition, graphene can improve the mechanical properties by generating grain refinement of Al. Therefore, it was confirmed that the GO content increased to 0.3 wt.% and a value of 129.33 MPa was measured for the hardness (Figure 6b). However, when the GO content increased to more than 0.4 wt.%, hardness decreased. In Figure 2f,g, it can be seen that GO was exfoliated from the Al particles. This problem decreases the density and mechanical properties by forming pores in the composites during the sintering process. Therefore, Al composites uniformly coated with GO can be lighter and harder than pristine Al bulk.

The specific heat and thermal diffusivity were analyzed by the laser flash method at room temperature. Thermal conductivity was calculated using the following equation with the measured density(α), specific heat (C_p_) and thermal diffusivity (p).

(1) $$k=a\times p\times C_{p}$$

**Figure 7:**
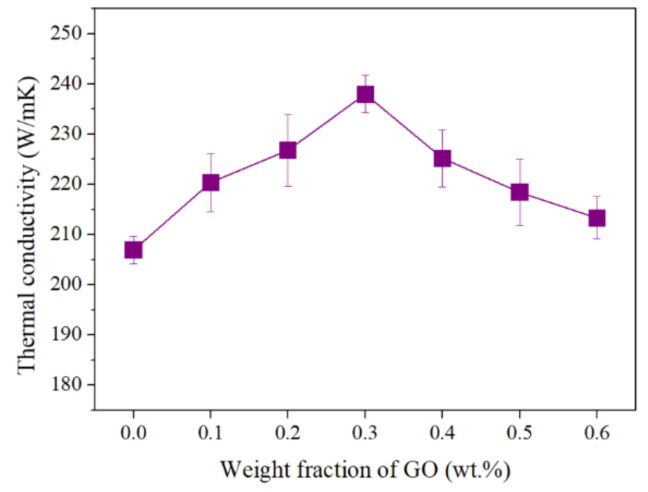
Thermal conductivity of GRAMCs with GO contents of 0 wt.% to 0.6 wt.%.

The graph of all calculated thermal conductivity is shown in Figure 7. The result of Al specimens without addition of GO was 206.9 W/mK. However, the Al/GO pellet containing 0.3 wt.% of GO had a thermal conductivity that was about 15% improved, showing a value of 238.0 W/mK.

## 4. Conclusions

In this work, the characteristics of GRAMCs with thermal conductivity improvements according to GO content were studied. First, 0.1 wt.% to 0.6 wt.% of GO coated Al particles were prepared using a pH-controlled self-assembly method to uniformly disperse GO in the Al matrix. Through SEM, XPS and Raman spectroscopy, it was confirmed that the GO was well-coated on the Al surfaces of the prepared Al/GO powders. Then, using SPS to minimize the formation of Al_4_C_3_, sintering was performed for a short period of 5 min at a temperature of 600 °C. After this, the densified composites were heat treated for 2 h at a temperature of 550 °C to improve the thermal conductivity through grain growth. The GRAMCs showed values of Al theoretical density of more than 99% due to the uniform dispersion of GO in the Al matrix by the self-assembly coating method. In cases of composites having GO content of 0.3 wt.%, the Vicker’s hardness increased by 47% and the thermal conductivity was improved by 15% compared to those values of bare Al. GRAMCs with GO coating on Al particles are expected to be applicable to high-strength and lightweight heat radiating applications; this mechanism and method can be applied to the development of various metal-nano carbon hybrid materials. We improved the dispersibility of Al/GO composites by GO coating on surface of Al particles. We finished by comparing the characteristics according to the content of GO. In the future, if the layer of GO coated on the Al powder is controlled as a monolayer, the properties of GRAMCs can be further improved. In addition, the coated GO still has many oxygen functional groups, is thermally decomposed in the sintering process, and maintains the potential to decrease the density of composites by making pores. The generated pores act as a factor to decrease the properties of the composite materials and eliminate the oxygen groups, it is therefore expected to be possible to manufacture GRAMCs with better thermal conductivity.

## Figures and Tables

**Table 1:** Curve fitting results of XPS C1s spectra of the GO and the Al/GO 0.3 wt.%.

GO C1s	Area (%)	Binding Energy (eV)	Al/GO C1s	Area (%)	Binding Energy (eV)
sp2 C-C	15.97	284.56	sp2 C-C	23.03	284.25
sp3 C-C	22.34	285.01	sp3 C-C	22.20	284.97
C-O	42.82	286.93	C-O	38.69	286.58
C=O	8.70	287.65	C=O	4.57	287.66
O-C=O	10.17	288.72	O-C=O	11.51	288.82

**Table 2:** Comparison of the mechanical and thermal properties of all the samples.

Samples	Bulk Density (g/cm^3^)	Related Density (%)	Vicker’s Hardness (MPa)	Specific Heat (J/gK)	Thermal Diffusivity (mm^2^/s)	Thermal Conductivity (W/mK)
Raw Al	2.699	99.93	91.83	0.899	85.27	206.9
Al/GO 0.1 wt.%	2.694	99.78	107.67	0.907	90.18	220.4
Al/GO 0.2 wt.%	2.696	99.85	117.80	0.916	91.85	226.8
Al/GO 0.3 wt.%	2.693	99.74	129.83	0.922	95.87	238.0
Al/GO 0.4 wt.%	2.685	99.44	123.50	0.928	90.36	225.2
Al/GO 0.5 wt.%	2.686	99.48	114.99	0.935	86.95	218.4
Al/GO 0.6 wt.%	2.677	99.15	104.50	0.938	84.94	213.3
